# The Role of Glial Cells in Regulating Feeding Behavior: Potential Relevance to Anorexia Nervosa

**DOI:** 10.3390/jcm11010186

**Published:** 2021-12-30

**Authors:** Linda Frintrop, Stefanie Trinh, Jochen Seitz, Markus Kipp

**Affiliations:** 1 Institute of Anatomy, Rostock University Medical Center, 18057 Rostock, Germany; markus.kipp@med.uni-rostock.de; 2 Institute of Neuroanatomy, RWTH Aachen University, 52074 Aachen, Germany; ntrinh@ukaachen.de; 3 Department of Child and Adolescent Psychiatry, Psychosomatics and Psychotherapy, RWTH Aachen University, 52074 Aachen, Germany; jseitz@ukaachen.de

**Keywords:** anorexia nervosa, glia cells, astrocyte, hypothalamus

## Abstract

Eating behavior is controlled by hypothalamic circuits in which agouti-related peptide-expressing neurons when activated in the arcuate nucleus, promote food intake while pro-opiomelanocortin-producing neurons promote satiety. The respective neurotransmitters signal to other parts of the hypothalamus such as the paraventricular nucleus as well as several extra-hypothalamic brain regions to orchestrate eating behavior. This complex process of food intake may be influenced by glia cells, in particular astrocytes and microglia. Recent studies showed that GFAP^+^ astrocyte cell density is reduced in the central nervous system of an experimental anorexia nervosa model. Anorexia nervosa is an eating disorder that causes, among the well-known somatic symptoms, brain volume loss which was associated with neuropsychological deficits while the underlying pathophysiology is unknown. In this review article, we summarize the findings of glia cells in anorexia nervosa animal models and try to deduce which role glia cells might play in the pathophysiology of eating disorders, including anorexia nervosa. A better understanding of glia cell function in the regulation of food intake and eating behavior might lead to the identification of new drug targets.

## 1. Introduction

In the central nervous system (CNS) exist billions of cells with various functions, molecular structures, and morphologies. These cells build up complex circuits to fulfill a specific task in the human body, i.e., eating behavior. However, the circuits are not limited to neuronal cells, also glia cells play an important element in these pathways. Recent studies showed that glia cells are active and important regulators in food intake behavior and are, thus, potential drug targets for metabolic disorders. Therefore, we summarize in this review the neuronal circuits of eating behavior in the hypothalamus, the role of glia cells in this process, and the potential influence of glia cells for eating disorders with a specific focus on anorexia nervosa (AN).

## 2. Neuronal Control of Appetite in the Hypothalamus

The regulation of food intake is one of the most complex behavioral processes in the CNS and we are still far away from entirely understanding its complexity. While a balance between energy intake and expenditure results in body weight maintenance, insufficient food intake for a longer period of time results in body weight loss, which can be severe (reviewed in [1]). Afferent signals from the brain stem and the gut signal to the hypothalamus, more specifically to the arcuate nucleus (ARC). These signals also include strong signals from the lateral septum which is itself connected to cortical networks [2]. As an example, a top-down pathway was revealed from the medial prefrontal cortex to neurons of the septum, which regulates food intake behavior in the hypothalamus [3].

**Figure 1:**
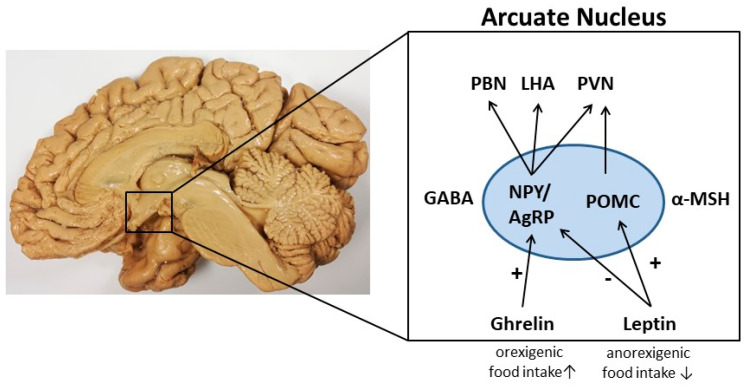
A schematic illustration of the control of food intake in the arcuate nucleus (ARC) of the hypothalamus. In this nucleus, two subpopulations of neurons, the orexigenic NPY/AgRP neurons, and anorexigenic POMC neurons, regulate the process of food intake. These neurons communicate with other hypothalamus nuclei such as the paraventricular nucleus (PVN), the lateral hypothalamic area (LHA), and the parabrachial nucleus (PBN). As metabolic hormone leptin can signal to the ARC by inhibiting NPY/AgRP neurons leading to reduced food intake. Furthermore, leptin activates POMC neurons resulting in decreased food intake. In contrast, ghrelin stimulates NPY/AgRP neurons and signals to orexigenic pathways. GABA, gamma-aminobutyric acid; MSH, melanocyte-stimulating hormone.

Several peripheral mediators exist which influence neuronal network activities with leptin, ghrelin, insulin, and glucose being the most widely studied. Leptin often referred to as the “satiety hormone”, is produced by adipocytes, proportional to fat levels (reviewed in [4]). Higher leptin concentration leads to an increased sense of satiety and, as a result, to a decrease in food consumption and vice versa (reviewed in [5]). By this mechanism, leptin maintains fat levels within a relatively narrow range. Leptin regulates fat levels by three distinct mechanisms: firstly, by decreasing food intake, secondly by increasing the body’s metabolic rate, and finally by increasing the body temperature. Leptin can reach the main region in which it operates the hypothalamus, which contains cells expressing leptin-receptors, by the bloodstream. When leptin binds to its receptor, satiety sets in and food intake decreases. More specifically, leptin inhibits neuropeptide Y (NPY)/agouti-related peptide (AgRP) neurons whilst simultaneously activating pro-opiomelanocortin (POMC) neurons (Figure 1). Appetite-enhancing (orexigenic) NPY/AgRP and appetite-suppressing (anorexigenic) POMC neurons are the major neuron populations in the ARC of the hypothalamus regulating food intake [6]. These neurons signal to diverse hypothalamic and extra-hypothalamic brain regions to orchestrate feeding and non-feeding-related behaviors.

Another peptide hormone regulating food intake is ghrelin synthesized in the hypothalamus and endocrine cells of the gastric mucosa. Its effects are antagonistic to those of leptin [7]. Ghrelin is the only known peripherally derived orexigenic hormone to increase appetite and subsequent food intake. Ghrelin levels drop shortly after a meal and increase before a meal and during fasting periods. In comparison to leptin, ghrelin stimulates the activation of NPY/AgRP neurons in the ARC of the hypothalamus and the release of appropriate orexigenic peptides and neurotransmitters [8,9]. In the next sections, we aim to provide a brief overview of the regulation of food intake by NPY/AgRP and POMC neurons.

## 3. Orexigenic Neuropeptide Y (NPY)/Agouti-Related Peptide (AgRP) Neurons

A subset of neurons located in the ARC of the hypothalamus synthesize NPY and AgRP. The central function of these neurons is to stimulate food intake. Accordingly, NPY/AgRP neuronal activity is elevated when the body is in a state of energy deficit [10]. Acute activation of NPY/AgRP neurons rapidly and dramatically induces food intake, decreases energy expenditure, and increases fat stores.

While the food intake regulating the function of NPY/AgRP neurons is well documented, there is evidence that eating behavior is also regulated by these neurons. Eating behavior includes feeding practices, food choice and motives, dieting, and eating-related problems. Activating only AgRP neurons substantially increased motivation for eating and drove intense food-seeking behavior, demonstrating that these neurons also orchestrate other complex behaviors in adult mice [11]. When food is accessible, the artificial activation of AgRP neurons enhances food consumption. In the absence of food, the same stimulus increases stereotypic and compulsive behavior [12]. Since food preferences and food-seeking behavior are conditioned processes, the ability of AgRP neurons to directly influence learning in mice has been examined: AgRP neurons predictively encoded the receipt of food by rapidly reducing neuronal activity, and this process involves learning [13]. In comparison, ablation of AgRP neurons in activity-based anorexia (ABA; see later in this review article) animal model led to reduced exercise and death of the mice while daily activation of these neurons increased compulsive running with no mortality indicating the neurons’ ability to regulate running behavior and survival [14]. Since inhibition of these neurons led to the inability to use fuels during food-restriction-associated exercise, it appears that AgRP neuronal circuits are connected to the regulation of food intake during starvation.

**Figure 2:**
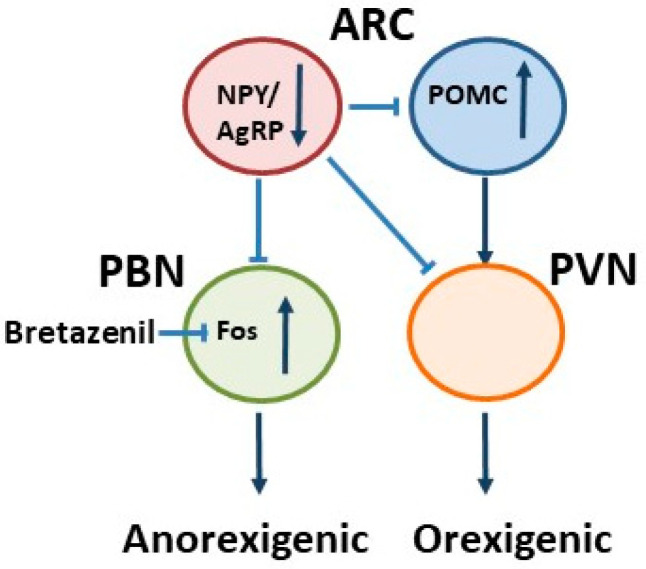
A schematic illustration of food intake regulation in the ARC when AgRP neurons are removed. As a result, starvation or anorexia occurs, while a direct application of bretazenil (a GABA_A_ receptor partial agonist) into the parabrachial nucleus (PBN) protects from starvation in adult mice. Modified from Wu et al., 2009. PVN, paraventricular nucleus.

It is a matter of debate which factors mediate the orexigenic function of the NPY/AgRP neurons. The inactivation of the genes encoding NPY, AgRP, or both had little effect on body weight regulation, suggesting that something else produced by these neurons does regulate their orexigenic function [15]. Since AgRP neurons mainly express gamma-Aminobutyric acid (GABA) [16], it is assumed that GABA may be the critical transmitter operant in AgRP neurons. Supporting the assumption that GABA is a key neurotransmitter regulating AgRP-induced food consumption, AgRP ablation led to the activation of second order neurons potentially due to the loss of GABAergic inhibition. In line with the orexigenic function of AgRP neurons, their selective ablation by the action of diphtheria toxin in mice resulted in starvation and elevated neuronal *Fos* expression as well as gliosis in the ARC and postsynaptic brain regions [17,18]. Beyond, in these animals, chronic subcutaneous application of bretazenil (a partial GABA_A_ receptor agonist) suppresses *Fos* activation and maintains food intake (Figure 2). The direct administration of bretazenil into the parabrachial nucleus (PBN), a direct target of AgRP neurons, was found to be necessary to sustain food intake. Thus, the inactivation of GABA biosynthesis in the ARC or the blockade of GABA_A_ receptors in the PBN in mice promotes anorexia. Therefore, the modification of GABA neurotransmitters released by AgRP neurons can influence food intake and, in turn, result in starvation. Beyond, it has been demonstrated that the selective inactivation of the vesicular GABA transporter gene (*Vgat*) led to a lean phenotype and a resistance to diet-induced obesity in mice [19]. An additional link for GABA functions in feeding-related starvation processes was revealed by Aoki et al. who showed that hippocampal GABA_A_ receptor subunits are increased in an animal model for AN [20]. Thus, this alteration in ABA animals is maybe be involved in the inhibition of hippocampal neuronal circuits and anxiety.

## 4. Anorexigenic Pro-Opiomelanocortin (POMC) Neurons

Another main population in the ARC of the hypothalamus are POMC neurons, in which the precursor protein pro-opiomelanocortin (POMC) produces many biologically active peptides via a series of enzymatic steps in a tissue-specific manner, including melanocyte-stimulating hormones (MSHs), corticotrophin (ACTH), and β-endorphin. These peptides play a role in an array of biological activities in the central and peripheral nervous systems (reviewed in [21]), however, the central function of POMC-derived peptides in the ARC is to inhibit food intake. During a meal, activation of POMC neurons results in the release of several POMC-derived peptides including α-MSH, which gradually promotes the onset of satiety and increased energy expenditure (reviewed in [21]).

In terms of eating behavior, the sensory detection of food is sufficient to rapidly reverse the activation state of these neurons induced by energy deficit [22] suggesting a central role in controlling eating behaviors such as foraging. In addition, POMC neurons might play a role in eating disorders since POMC deficiency results in obesity [23]. We currently know that the complex process of food intake is not only regulated by neurons but also by glia cells such as astrocytes, which can actively influence these signaling cascades [24].

## 5. Astrocyte Pathology

Glia cells have long been considered passive cells which support the mechanical stability of neuronal networks. These cells are currently recognized as active regulators of neuronal function and are therefore able to shape different types of behavior such as learning and memory (reviewed in [25,26]). In the CNS, glia cells are divided into astrocytes, microglia, ependymal cells, and oligodendrocytes while only the first two seem to be involved in the cascades of food intake.

In pathological conditions e.g., after CNS injury, reactive astrogliosis triggered by cytokines (e.g., TGF-α, CTNF, and IL-6 [27]) is induced and accompanied by morphological and functional changes which influence disease progression and recovery processes (reviewed in [25,28,29]). Besides the role in astrogliosis, astrocytes influence the development, maintenance, and function of the blood-brain barrier and thereby control the influx and efflux of various metabolites. Their ability to sense as well as transport nutrients (e.g., glucose and lipids) and hormones (e.g., leptin and insulin) from the bloodstream into the brain suggest that they play a key role in metabolism [30,31]. Since neurons are not able to store vast amounts of energy, astrocytes provide neurons with energy equivalents, a supportive astrocyte function that might be disturbed in conditions of starvation [30]. Thus, astrocytes would be able to worsen an energy-deficient condition, e.g., by an impaired glucose transport of hypothalamic astrocytes to neurons [32,33,34].

In conclusion, knowledge about the physiology of astrocytes creates opportunities to identify new targets to treat diseases including eating disorders. We now address the influence of astrocytes in food intake processes because these cells are subject to change in the ABA animal model [35,36].

## 6. Disturbed Gliotransmission as a Regulator of Feeding Behavior

**Figure 3:**
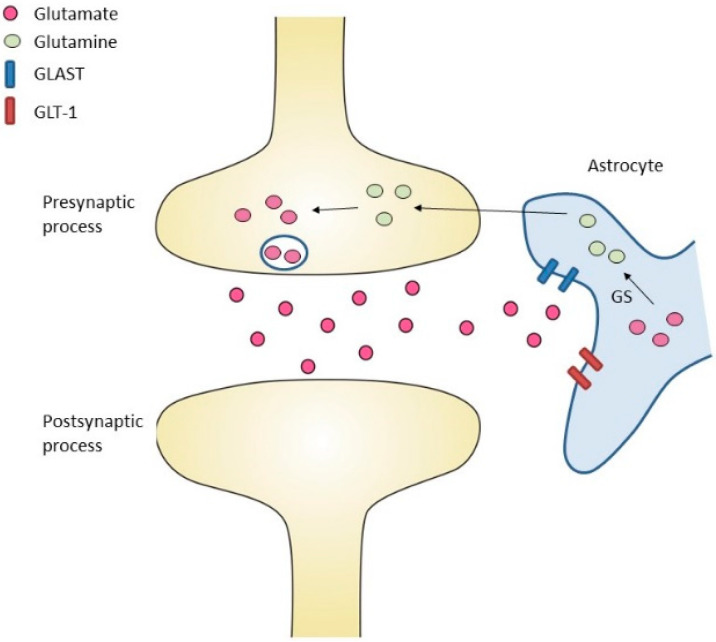
The role of astrocytes in glutamate uptake. Glutamate is released into the synaptic cleft. Glutamate transporter 1 (GLT-1) and L-glutamate/L-aspartate transporter (GLAST) transfer glutamate into astrocytic cells. Glutamine synthetase (GS) converts glutamate into glutamine. Glutamine is released from astrocytes via specific transporters and then taken up into neurons. In the neuron, glutamine is converted to glutamate.

It has been shown that astroglia in vitro directly signals to neurons and other astroglia through Ca^2+^-dependent exocytosis of neurotransmitters [37,38]. Subsequently, the term “gliotransmission” was created (i.e., active information transfer from glia to neurons) to describe this phenomenon (reviewed in [39]). Today it is broadly accepted that astrocytes receive neuronal information via a wide range of membrane receptors and other sensory mechanisms and translate this information into a complex intracellular Ca^2+^ code and other signal-transduction pathways (reviewed in [40]). Theoretically, gliotransmission may be possible via different mechanisms including SNARE (N-ethylmaleimide-sensitive factor attachment protein receptor)-mediated vesicular release, channel- and transporter-mediated mechanisms [41] as well as changes in neurotransmitter and ion uptake by astrocytes [42] from the synaptic cleft. Astrocytes are therefore able to (i) control presynaptic transmitter release probability, (ii) shape postsynaptic excitability, (iii) regulate different forms of activity-dependent and tonic synaptic plasticity as well as (iv) influence complex oscillatory network states via gliotransmission (reviewed in [40]). These essential functions of astrocytes in gliotransmission can be interfered with by glutamatergic signaling cascades: An important task of astrocytes is to take up, metabolize and recycle glutamate that is released into the synapse (Figure 3). When glutamatergic neurons are excited, glutamate is released into the synaptic cleft and binds to post-synaptic receptors for signal propagation. The remaining glutamate needs to be removed to terminate the signal. The specialization of astrocytic endfeet enables these cells to remove neurotransmitters rapidly and efficiently from the synaptic cleft to maintain homeostasis. After uptake, which is mainly mediated by Na^2+^-dependent transporters L-glutamate/L-aspartate transporter (GLAST) [43] and glutamate transporter 1 (GLT-1) [44], glutamine synthetase (GS) amidates glutamate to glutamine (GLN). As a result, glutamine is released from astrocytes via specific transporters and then taken up by neurons (Figure 3). In the neuron, glutamine is deamidated by phosphate-activated glutaminase (PAG) to glutamate, which completes the glutamate-glutamine cycle.

Following the hypothesis that a dysfunctional glutamatergic gliotransmission system may influence behavior, it has been shown that some extrasynaptic N-methyl-D-aspartate (NMDA) receptors antagonists drive antidepressive effects in animal models of depression [45]. Furthermore, there are hints that gliotransmission regulates eating behaviors, i.e., due to the neurotransmitter receptors G protein-coupled receptor metabotropic glutamate receptors subtype 5 (mGluR5) which is expressed by astrocytes and is important for gliotransmission [46]. The administration of memantine, an uncompetitive NMDA receptor antagonist, and 3-((2-Methyl-4-thiazolyl)ethynyl)pyridine (MTEP), an allosteric metabotropic mGlu5 receptor antagonist, decreased food consumption in a baboon model of binge-eating disorders [47]. After food deprivation for one night, mGluR5^−/−^ mice ate significantly less than mGluR5^+/+^ controls when both groups were refed [48]. However, since mGluR5 expression is not restricted to astrocytes but also found in neurons [49], it remains to be clarified whether these observations are specific to astrocytes although several hints suggest that glutamatergic gliotransmission plays a role in food intake control. Additionally, changes in glutamatergic signaling in an experimental ABA animal model were found: The expression of the α-amino-3-hydroxy-5-methyl-4-isoxazolepropionic acid (AMPA) receptor subunits GluA1, which mediates together with other subunits synaptic transmission, is enhanced in the nucleus accumbens potentially due to a disturbed mesocorticolimbic reward circuitry [50]. A recently published study using the same model showed that the increase in running wheel behavior correlates negatively with GLT-1 levels in the hippocampus indicating GLT-1 enhancement may counteract the severity of AN [51]. In summary, there is good evidence that glutamatergic signaling plays an important role in starvation processes. Further, astrocytes play an interesting role in behavioral disorders.

## 7. Astrocytes in Behavioral Disorders

Astrocytes are strategically located around neurons and synapses and can, due to the expression of neurotransmitter receptors on their membranes, sense neuronal activity. In response, astrocytes release neurotransmitters or co-transmitters such as glutamate, GABA, D-serine, or adenosine triphosphate (ATP) which can, as a result, influence neuronal activity. Assuming there is reciprocal communication between astrocytes and neurons, scientists started examining the possible role astrocytes may have in regulating behavior. Studies in animals have shown that astrocyte interaction with synapses occurs under physiological and pathological conditions, and this indeed shapes behavioral responses [52,53,54]. For example, chemogenetic activation of astrocytes in relevant circuits affected memory performance [53,55] and activation of astrocytes by DREADD (designer receptors exclusively activated by designer drugs) located in the medial central amygdala led to the extinction of learned fear memory in a cued fear conditioning task [55]. Furthermore, astrocytes also play a role in emotion and mood regulation, thus contributing to the development of depressive-like behavior. After the administration of an astrocyte-specific toxin into the prefrontal cortex, focally astrocyte-depleted animals showed anhedonia, anxiety-like behavior, and helplessness during the forced swim test [56]. Another study demonstrated that multiple endocrine neoplasia type 1 (Men1) expression is attenuated in the brain of mice exposed to chronic unpredictable mild stress, and astrocyte-specific reduction of Men1 leads to depressive-like behavior [57]. Interestingly, Men1 deletion in astrocytes enhanced pro-inflammatory pathways, linking depressive behavior to astrocyte-mediated inflammation. Numerous other studies have associated astrocyte dysfunction with the pathogenesis of major depressive disorders (reviewed in [58,59]), including the notion that the density of GFAP-immunoreactive astrocytes was decreased in the hippocampus of patients suffering from major depressive disorders [60]. Furthermore, glial ablation in the prefrontal cortex led to depressive-like symptoms in rats [56], suggesting that the loss of glia cells accompanies depressive symptoms. In summary, these data show that astrocytes are key regulators of behavior, and astrocyte dysfunction may contribute to the development and progression of behavioral disorders.

In the next section, we will discuss to what extent peripheral mediators of feeding behaviors, in particular leptin, exert their effects via modulation of astrocyte function.

## 8. Astrocytes as Targets of Peripheral Food Intake Signals

As already outlined above, the “satiety hormone” crosses the blood-brain barrier (BBB) through a selective transport system [61] and binds to its receptors which are, of note, not only expressed by neurons but also by astrocytes. There is good evidence that the interaction of leptin with astrocytes is functionally relevant. Conditional deletion of the astrocytic leptin receptors not just altered glial morphology but synaptic inputs onto hypothalamic neurons that are involved in feeding control as well [62]. Leptin-regulated feeding was diminished, whereas feeding after fasting or ghrelin administration was elevated in mice with astrocyte-specific leptin receptor deficiency. Furthermore, in a similar model of astrocyte-specific leptin receptor knockout mice, the leptin-induced activation of signal transducer and activator of transcription 3 (pSTAT3) signaling in the hypothalamus was reduced [63]. As a result, following a high-fat diet, mild gliosis and greater rise of fat mass were observed indicating that the influence of leptin is mediated at least in part via astrocytes [63]. Importantly, astrocytic leptin receptors also modulated the effectiveness of selective serotonin reuptake inhibitors in depression [64], suggesting an additional crosstalk between leptin and other neurotransmission circuits. Interestingly, one study has shown that astrocytic leptin receptors are involved in shaping glutamatergic gliotransmission in the hippocampus [65]. Whether a similar mechanism exists in food intake-related neuronal circuits in the hypothalamus remains to be determined. For a summary of the effects of leptin on glial cells, we like to refer to a recently published review article [66].

In comparison to leptin-mediated peripheral food intake signals, astrocyte function appears to interfere with the appetite-stimulating hormone ghrelin (reviewed in [67]). Astrocyte stimulation in the medial basal hypothalamus reduced basal- and ghrelin-involved food intake [68]. This reduction in food intake was mediated by the inactivation of AgRP neurons through adenosine A1 receptors in the ARC indicating that glia cells can sense metabolic conditions and play an important role in food intake control by orchestrating hypothalamic function. Furthermore, astrocytic insulin receptors are necessary for glucose and insulin entry into the brain and by this mechanism can regulate the glucose-dependent activation of POMC neurons [69]. Hypothalamic glia cells seem to regulate glucose sensing by insulin signaling further highlighting that astrocytes can regulate food intake.

Chemogenetic astrocyte activation in the dorsal vagal complex indicated that these cells could decrease food intake when activated and are associated with the integration of peripheral satiety pathways [70]. Following a high-fat diet, GFAP^+^ cell number increased, and these cells developed a more complex morphology indicating that astrocytes respond to nutritional changes. A mechanism due to an additional specialized astrocytic subtype in the hypothalamus, the Gomori-positive astrocyte, is also possible (reviewed in [71]). This subtype is abundant in the ARC, expresses GLUT2, and can increase glucose-dependent oxidative metabolism suggesting the relevance of Gomori-cells in energy signaling [72]. In conclusion, astrocytes are targets of peripheral food intake signals which are associated with leptin-, ghrelin- and insulin-mediated signals and should be further explored.

Besides astrocytes, microglia (another type of glia cell) have been shown to influence food intake by regulating signaling cascades, and this cell type will be discussed in the following Section.

## 9. Microglia Pathology

Microglia cells were recognized as a specific cell entity more than a century ago [73]. Since then, our knowledge of their function has greatly improved. One of their main functions as phagocytes is to remove cell debris, for example after injury of the CNS. Today we know that the role of microglia is more complex, especially in regulating functional and synaptic plasticity in the CNS (reviewed in [74]). Under physiological conditions, microglia are able to express signaling molecules that change synaptic transmission and in turn synaptic plasticity (reviewed in [75]).

Following inflammation, the morphology of microglia changes dramatically into an amoeboid morphology with retracted processes, and they are also capable of regulating plasticity by modifying synaptic connections [76]. Once activated, a process is known as microgliosis, microglia can differentiate into M1 (inflammatory) or M2 (immunosuppressive) phenotypes (reviewed in [77]). This dynamic switch from M2 to M1 is related to neurodegenerative disorders, obesity, and insulin resistance. Moreover, in animal models of obesity, the hypothalamic microglia have been shown to display a morphologically activated phenotype [78], suggesting that microglia are involved in inflammation-mediated signaling cascades as a result of excessive food intake.

In terms of glia cell interaction, it is known that astrocytes and microglia communicate with one another and, as a result, astrocytes influence microglia function and morphology, and vice versa (reviewed in [79]). The complex interplay of glia cells can regulate neuronal functions and therefore contribute to the regulation of feeding-related circuits.

## 10. Neuroinflammation in Eating Disorders and the Role of Microglia

The involvement of inflammation and microglia cells in the complex processes of food intake regulation is not completely clarified, however, our understanding is constantly growing. The results of several studies suggest that excessive food intake results in microgliosis [80,81,82]. After treatment with HFD, microglia density in the ARC has been found to be increased, whereas, in mice lacking leptin receptors, anti-IBA1 immunoreactivity was decreased [80]. The central application of the antimitotic drug arabinofuranosyl cytidine blocked HFD-induced cell microglia proliferation [81], paralleled, on a functional level, by an ameliorated development of obesity. Therefore, microglia influence food intake signaling pathways, further emphasizing that these cells are a potential target for interventions in patients with eating disorders. Due to their phagocytic function, microglia sense pathogen-associated molecular patterns via toll-like receptors (TLRs). TLR-2 activation in the hypothalamus following an injection of its ligand Pam3CSK4 induced anorexia nervosa-like symptoms in mice, such as body weight loss. This is associated with activation of microglia in the ARC and enhanced activity of POMC neurons indicating that microglia can regulate food intake pathways by TLRs [83]. However, these starvation symptoms can be due to general sickness as a result of the injections and therefore do not represent a good AN model. In a murine model lacking leptin receptors in myeloid cells including microglia, body weight was increased, and reduced POMC neuron numbers in the ARC and a-MSH projections from the ARC to the PVN were found [84]. Therefore, microglia are involved in leptin-mediated changes in food intake regulation.

To sum up, there is good evidence that both glia cell populations, astocytes as well as microglia, are important entities to mediate food intake pathways, potentially also in nutrient-deficient conditions as in eating disorders like AN.

## 11. Anorexia Nervosa (AN)

AN is characterized by body image disturbances and significant weight loss [85]. AN has the highest mortality rate of any mental disorder [86] and is the third most common chronic disease in adolescence and young adulthood, particularly affecting females [87].

In terms of neuropathology, AN is associated with extensive brain volume changes. Two meta-analyses have demonstrated in detail that gray matter volume is decreased by 6% and white matter volume by 4% in patients with acute AN (reviewed in [88]). These findings are preliminary as there are differences in the replications between studies (reviewed in [89]). Furthermore, brain volume loss has been associated with neuropsychological deficits, such as impairments in logical thinking and visuospatial memory [89,90,91,92]. Moreover, gray matter volume reduction has been found to positively correlate with an increased drive for thinness [93]. Several studies have shown that following long-term body weight restoration, brain volume reductions may be reversible [89,94,95,96]. However, it remains unknown whether there are long-term complications or residues, especially in severely and chronically ill patients with AN [97].

The cellular changes that underlie this brain volume reduction in patients with AN are mostly unclear. Dehydration as the sole reason for brain volume loss seems unlikely due to normal serum and normal to low urine osmolarity [97]. Therefore, an important new focus for research is to analyze the neuropathological processes in patients with AN underlying brain atrophy. In principle, loss of brain volume might be due to the destruction of virtually every neuronal element including axons, synapses, myelin sheets, or glia cell bodies. Lately, serum GFAP and neurofilament light chain (NF-L) levels were increased in the patients compared to healthy controls and decreased upon short-term partial weight restoration, potentially indicating neural and glial damage during starvation [98]. Post-mortem analyses of patients with AN demonstrated reduced spine density as well as a changed spine morphology, which was paralleled by gliosis in periventricular brain regions and the ventromedial hypothalamus [99,100,101]. Furthermore, alterations in neuropil [102], synapses [103], and glial cells [36] have been reported in animal models of AN or starvation. A few independent studies identified altered glia cells in the brain in the models for AN suggesting that these cells underlie and/or worsen the pathophysiology of the disease, for example by a disturbed astrocytic glucose supplementation for neurons [35,104,105,106]. To investigate the complex signaling cascades of food intake and the relevant influence of glia cells, eating disorder animal models (e.g., AN models) are of great importance and, thus, are reviewed in the following Section.

## 12. Glia Cell Pathology in AN

Animal studies have given first insights into the underlying pathophysiology of AN. The ABA animal model mimics the most important pathological parameters of patients with AN such as amenorrhea, bodyweight reduction, hypothermia, hypoleptinemia, and hyperactivity. The first ABA model consequently called the original model, combines a period of *ad libitum* food intake not longer than 1–3 h per day, combined with free access to a running wheel [107]. Paradoxically, the animals run voluntarily not only during starvation but also during feeding times. Some animals, therefore, run themselves to death. This extensive running behavior can be interpreted as food-seeking or compulsive behavior [14]. Interestingly, the hyperactivity was reduced by administrating leptin, indicating a prominent role of leptin for activity regulation [108]. In this model, mice run in the hours before the food is available. The enhancement of running wheel activity prior to the feeding periods is called food-anticipatory activity (FAA) [109,110]. In the ABA model, this FAA correlated with the susceptibility for bodyweight loss and, only for resistant animals to bodyweight loss, resulting in enhanced food intake. Thus, hyperactivity can be used as a predictive tool for the severity of ABA [111].

Some studies addressed the important question of whether the brain atrophy observed in patients with AN can be mimicked in the ABA model and which cellular alterations underlie this brain atrophy under conditions of food restriction. To analyze chronic food restriction, our group established a slightly modified ABA model that avoids the high mortality rate observed in the original model, whilst including a fixed level of body weight loss maintained over a longer period of time. The start of the modified ABA model is characterized by an acute starvation phase, in which the rats received 40% of their baseline food intake until a 25% weight reduction is reached. Afterward, a period of chronic starvation is maintained by holding the animals’ body weight at 75% of their initial body weight for two weeks by giving them just enough food to maintain this weight [112]. Thus, this ABA model can be assumed to more accurately represent the often-chronic course of AN.

**Figure 4:**
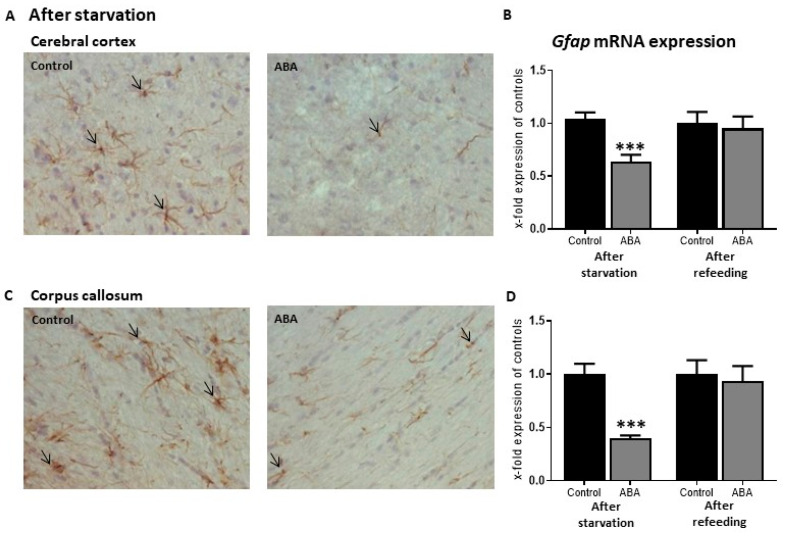
Effects of chronic starvation on GFAP^+^ cell densities in the cerebral cortex (**A**) and the corpus callosum (**C**) in activity-based anorexia (ABA) rat model. The arrows mark GFAP^+^ astrocytes. GFAP^+^ cell densities and mean mRNA expression of *Gfap* were reduced after starvation in the ABA group compared to the control group. After refeeding *Gfap* mRNA expression was reversed to control levels in both brain regions (**B**,**D**). *** *p* ≤ 0.001, two-sided student’s *t*-test. Modified from Frintrop et al., 2019.

In this model, we showed a marked reduction of GFAP^+^ astrocyte cell density in the white matter corpus callosum (CC) and the cerebral cortex (CX) of ABA rats in comparison to controls (Figure 4) [35,36]. The reduction of GFAP^+^ astrocyte cell density was accompanied by a reduction in the area covered by GFAP^+^ cells and *Gfap* mRNA expression in both brain areas. Furthermore, brain volume reductions of 6 and 9% respectively were found in the same brain regions of ABA animals which is in line with findings in patients with AN [99,113]. No changes in neuron or oligodendrocyte cell density were found, suggesting an astrocyte-specific effect. Of note, after acute starvation, no brain (pseudo-) atrophy has been found [35]. It has been suggested that immature cells present in the CC of mice continue dividing throughout life and their progeny give rise to astrocytes and oligodendrocytes [114]. Reduced astrocyte densities after chronic starvation could be either due to astrocyte degeneration or a reduction of astrocyte turnover. After chronic starvation, cell proliferation (examined with the marker Ki67) was reduced by 50% in CC and CX of ABA rats compared to normally fed rats while apoptosis-rate was not altered, pointing to a reduction of new cell production rather than increased cell death. Our findings are in line with Barbarich-Marsteller et al.’s results [115], who already observed a reduction in cell proliferation after starvation, identified by 5-bromo-2-deoxyuridine (BrdU) incorporation and anti-Ki67 immunohistochemistry in the hippocampus and CC. In our studies after refeeding (20 days of *ad libitum* food access), the starvation-induced effects were mainly reversible in the CX but not CC in ABA animals (Figure 4) [36]. Although we don’t know the basis for this observed regional difference, it can be speculated that white matter needs longer to regenerate than gray matter. In contrast to our observation following refeeding a study recently published by Hurley et al. revealed a decrease in astrocyte density in the medial prefrontal cortex in prone ABA animals in comparison with resistant ones [104]. The difference to our study is maybe due to the different selection of groups (prone, resistant animals vs. ABA, control), various starvation lengths (7 days vs. 3 weeks), different measurement methods (optical density vs. cell counting), different regions of the brain (Bregma +2.7 vs. −2.3) and/or refeeding length (10 vs. 20 days).

Besides the ABA model, a dehydration-induced anorexia (DIA) model also exists, in which the animals receive 2.5% NaCl solution to drink leading to dehydration and reduced body weight [110]. In line with our results, Reyes-Haro et al. have shown significant reductions in GFAP^+^ cell numbers in the white matter, mainly in the body of the CC, in rats with DIA. Interestingly, no alteration of average astrocyte size was demonstrated indicating that there is no global change in astrocyte morphology. Furthermore, research from the same group revealed that the GFAP^+^ astrocyte cell number in the hippocampus was significantly reduced [116]. Moreover, lower GFAP protein expression and increased expression of intermediate filaments (vimentin and nestin) in the DIA group were demonstrated. In summary, both anorexia rodent models (ABA and DIA) share the characteristic of GFAP^+^ cell number loss, underpinning an important regulatory function of astrocytes during food intake regulation.

Another consequence of starvation appears to be inflammation shown by an increase of IL-1β and IL-1R1 protein expression in the hypothalamus of ABA animals [117]. Following DIA, increased microglia cell densities have been found in the hippocampus [106], associated with higher IBA1, TNF-α, IL-6, and IL-1β protein levels, suggesting the presence of a neuroinflammatory environment in the hippocampus. Additionally, increases in anti-IBA1 positive microglia cell numbers were evident in the medial prefrontal cortex and orbitofrontal cortex of DIA rats [118].

The described changes on the cellular level are paralleled with behavioral deficits shown in ABA rodents, e.g., impaired memory performance in the novel object recognition (NOR) task [119] and reduced anxiety-like behavior in the elevated plus-maze test [120,121]. The impairment of NOR memory was reversed following administration of anti-NPY antiserum, Y5 receptor antagonist CPG71683, and anti-AgRP antibody connecting this behavior task with hypothalamic neuronal circuits [122]. Further, DREADD inhibition of the medial prefrontal cortex to the nucleus accumbens shell pathway led to improvements in cognitive flexibility and lower weight loss indicating that this pathway plays a role during food intake behavior [123].

Cell culture studies additionally gave interesting insights into glia cell pathology. An in vitro semi-starvation model in primary rat astrocytes has been established in our group by Kogel et al. [124]. The cells were treated with a low glucose concentration (2 mM) in the culture medium for 15 days to mimic chronic semi-starvation. Morphological alterations, as well as increased expression of the pro-inflammatory A1 phenotype markers, indicated increased reactivity of the astrocytes under glucose deprivation. Furthermore, expression levels of pro-inflammatory cytokines were found to be elevated. Increased expression of genes involved in the unfolded protein response (UPR) points towards the activation of cellular stress responses. Most of the observed effects were reversible after six days of culturing in a medium with normal glucose levels (i.e., 25 mM glucose). These observations further support the hypothesis that astrocytes are important players in the pathophysiology of food starvation.

## 13. Pathways of Food Intake in ABA

Consequently, another important question is how food intake-related pathways are changed due to starvation processes in the ABA model. A few studies have shown first insights into this topic: Increased Fos expression in the hypothalamus (ARC and supraoptic nucleus) indicates neuronal activation in ABA animals when compared with normally fed animals [125]. Furthermore, *Npy* and *Agrp* mRNA expression was upregulated in ABA animals while *Pomc* expression was reduced [126]. However, another study has shown an increased expression of *Pomc* mRNA level; this may be dependent on the length of starvation [127]. Furthermore, examination of the ventral medial hypothalamus revealed that melanocortin expression, a POMC cleavage product, was increased in ABA animals [128]. Surprisingly, intracerebroventricular administration of NPY as an orexigenic peptide in the ABA model led to decreased food intake but enhanced running wheel activity [126]. The administration of AgRP as an orexigenic peptide counteracted the suppression of food intake and reduced hyperactivity due to an improvement of symptoms in the starvation model [128]. Chronic treatment of ABA rodents with α-MSH, a cleavage product of POMC, worsened symptoms. This was evident by a reduced food intake, increased running wheel activity, and HPA axis activation [129]. To conclude, food intake mediating pathways are changed due to starvation in the ABA model suggesting that orexigenic and anorexigenic neuropeptides in the ARC are useful targets to promote eating behavior, even in pathological states. The finding on NPY administration and the role of these neuropeptides needs to be clarified in future research.

## 14. Conclusions and Outlook

**Figure 5:**
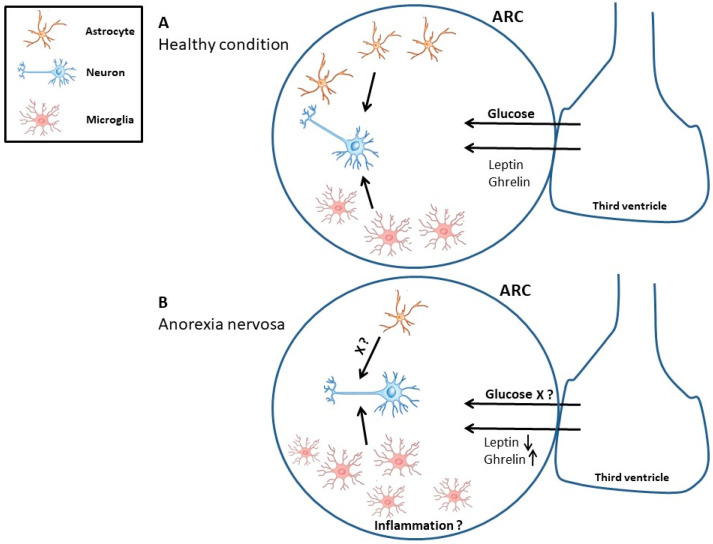
The interaction between neurons, microglia, and astrocytes in the control of food intake. Under healthy conditions, the regulation of food intake resembles a homeostatic interplay of glia cells and neurons (**A**). Astrocytes act for example as glucose sensors and influence plasticity through their contact with synapses. A hypothetic model demonstrates the starvation-induced effects in the hypothalamus in anorexia animal models, e.g., a decrease in astrocyte cell number and function and an increase in microglia cell number as a result of neuroinflammation (**B**). ARC, arcuate nucleus.

In summary, the brain volume reduction in the ABA model is linked to a reduction in GFAP^+^ cell density. We suggest that these cellular changes also occur in the ARC of the hypothalamus associated with astrocytic dysfunction and an inflammatory environment (Figure 5). The reasons for GFAP^+^ cell density loss could be reduced proliferation of astrocytic precursor cells, apoptosis/necrosis of astrocytes, decreased *Gfap* mRNA expression, and/or potentially due to UPR induction which might be linked to an inhibition of GFAP protein synthesis. Our observation of reduced overall cell proliferation suggests that disturbed astrocyte turnover plays an important role, a hypothesis that will be clarified in the future with immunofluorescence double staining experiments. Additionally, reduced astrocyte density can lead to disturbed astrocytic function which, in turn, could change behavior potentially explaining neuropsychological deficits in psychiatric disorders. Causal interactions between astrocytes and behavioral alteration in starvation need further exploration.

As previously described, also neurons play an important role in food intake control and behavior. For example, neuronal impairment can worsen the energy-deficient condition by changes in synaptic plasticity which can be investigated in animals with neuronal and/or astrocytic specific fluorescent protein expression.

In conclusion, experimental animal models for AN demonstrate the potential influence of glia cells in the neuropsychological progression of eating disorders. Astrocytes and perhaps microglia are therefore an important research area in the context of eating behavior to find potential new drug targets for interventions in patients with eating disorders, especially AN. Further research should focus on the interaction between astrocytes and neurons in the hypothalamus to analyze the complex interplay in food intake-related pathways.
